# Barriers and facilitators when implementing family involvement for persons with psychotic disorders in community mental health centres – a nested qualitative study

**DOI:** 10.1186/s12913-022-08489-y

**Published:** 2022-09-12

**Authors:** Kristiane Myckland Hansson, Maria Romøren, Reidar Pedersen, Bente Weimand, Lars Hestmark, Irene Norheim, Torleif Ruud, Inger Stølan Hymer, Kristin Sverdvik Heiervang

**Affiliations:** 1 Centre for Medical Ethics, University of Oslo, Postbox 1130 Blindern, 0318 Oslo, Norway; 2 Division of Mental Health Services, Akershus University Hospital, Sykehusveien 25, 1474 Nordbyhagen, Norway; 3 Center for Mental Health and Substance Abuse, Faculty of Health and Social Sciences, University of South-Eastern Norway, Drammen, Norway; 4 Faculty of Health Sciences, OsloMet Oslo Metropolitan University, Oslo, Norway; 5 Division of Mental Health and Addiction, Vestre Viken Hospital Trust, Drammen, Norway; 6 Institute of Clinical Medicine, University of Oslo, Oslo, Norway; 7 Early Intervention in Psychosis Advisory Unit for South East Norway, Division of Mental Health and Addiction, Oslo University Hospital, Oslo, Norway

**Keywords:** Family involvement, Family interventions, Family psychoeducation, Psychotic disorders, Implementation, Barriers, Facilitators, Mental health services research

## Abstract

**Background:**

The uptake of family involvement in health care services for patients with psychotic disorders is poor, despite a clear evidence base, socio-economic and moral justifications, policy, and guideline recommendations. To respond to this knowledge-practice gap, we established the cluster randomised controlled trial: Implementation of guidelines on Family Involvement for persons with Psychotic disorders in community mental health centres (IFIP). Nested in the IFIP trial, this sub-study aims to explore what organisational and clinical barriers and facilitators local implementation teams and clinicians experience when implementing family involvement in mental health care for persons with psychotic disorders.

**Methods:**

We performed 21 semi-structured focus groups, including 75 participants in total. Implementation team members were interviewed at the initial and middle phases of the intervention period, while clinicians who were not in the implementation team were interviewed in the late phase. A purposive sampling approach was used to recruit participants with various engagement in the implementation process. Data were analysed using manifest content analysis.

**Results:**

Organisational barriers to involvement included: 1) Lack of shared knowledge, perceptions, and practice 2) Lack of routines 3) Lack of resources and logistics. Clinical barriers included: 4) Patient-related factors 5) Relative-related factors 6) Provider-related factors. Organisational facilitators for involvement included: 1) Whole-ward approach 2) Appointed and dedicated roles 3) Standardisation and routines. Clinical facilitators included: 4) External implementation support 5) Understanding, skills, and self-efficacy among mental health professionals 6) Awareness and attitudes among mental health professionals.

**Conclusions:**

Implementing family involvement in health care services for persons with psychotic disorders is possible through a whole-ward and multi-level approach, ensured by organisational- and leadership commitment. Providing training in family psychoeducation to all staff, establishing routines to offer a basic level of family involvement to all patients, and ensuring that clinicians get experience with family involvement, reduce or dissolve core barriers. Having access to external implementation support appears decisive to initiate, promote and evaluate implementation. Our findings also point to future policy, practice and implementation developments to offer adequate treatment and support to all patients with severe mental illness and their families.

**Trial registration:**

ClinicalTrials.gov Identifier NCT03869177. Registered 11.03.19.

**Supplementary Information:**

The online version contains supplementary material available at 10.1186/s12913-022-08489-y.

## Background

The uptake of family involvement in health care services for patients with psychotic disorders is poor [1–3] despite a robust evidence base of decreased rates of relapse and hospital admissions, and better adherence with medication among patients [4–7]. Moreover, family interventions are shown to reduce psychological distress and care burden, and to improve family functioning and quality of life among relatives [8–10]. This knowledge-practice gap paradox results in patients and relatives being deprived of highly recommended treatment and support [11–19]. Among the most important factors to improve the outcome of schizophrenia is the translation of psychosocial treatments from research to the field [20]. Family psychoeducation (FPE), designed to engage, inform, and educate family members so that they can assist the person with severe mental illness in managing the illness, but also to reduce family distress and burden [18], is one such treatment. The scientific evidence of improvement in patient outcomes has been consistent [4, 5, 7], and research studies, policies, and guidelines have been calling for an increased uptake of FPE for decades [2, 4, 21, 22]. In addition, moral and socio-economic arguments to involve and support relatives in the context of deinstitutionalisation and subsequent emergence of community care strengthens this appeal [8, 9, 23]. In this study, the terms “family” and “relative” cover anyone who provides substantial and unpaid support to a person with psychotic disorder. The term “family involvement” comprises both a basic level of family involvement and support and more comprehensive family interventions, such as FPE.

Why is the implementation of family interventions like FPE this scarce, despite being recognised as essential treatment during all stages of psychotic disorders [2, 24]? Multifaceted problems of integrating new evidence‐based practices into usual care partly explain why several previous attempts to implement family interventions in routine care have failed [18]. The research literature suggests a lack of financial incentives and prioritisation, lack of managerial support, restricted access to training and supervision, lack of time, caseload size, and shortfall in staff resources as major system-level barriers [2, 25–28]. Factors that are particularly challenging when implementing family involvement include the lack of systems and structure for carrying out family involvement, and practical difficulties when attempting to realise family involvement [26, 28]. Furthermore, staff attitudes, organisational cultures and paradigms can hinder the uptake of family interventions, for example by leading to varying ownership, low confidence in that family involvement can be helpful, and that family involvement are considered secondary or optional [25, 26, 28]. Impediments at the clinical level include patients refusing to involve their relatives, patient confidentiality [26, 29], lack of therapist confidence and competence in conducting family involvement, and lack of families to work with [26]. Furthermore, different stakeholder groups often have contrasting perspectives regarding barriers, and lack of trust between stakeholders, in this case, patients, relatives and health care personnel, is considered a major challenge when collaborating with relatives [26].

To accelerate the implementation of family involvement in Norwegian mental health care, we established the IFIP study: Implementation of guidelines on Family Involvement for persons with Psychotic disorders in community mental health centres (CMHCs) [30]. The IFIP study is a cluster randomised controlled trial that aims to increase the uptake of recommendations on family involvement from the national guidelines in Norway [11, 12]. A key part of the implementation strategy was a so-called “whole-ward” or “whole-system” approach [28, 31]. The IFIP intervention consists of the following elements:

I Clinical interventions:
  1.1 A basic level of family involvement and support (BFIS).
  1.2 Family psychoeducation (FPE) in single-family groups.
II Implementation interventions:
  2.1 Training and guidance of health care personnel.
  2.2 A family coordinator.
  2.3 Other implementation measures.

The IFIP study protocol [30] provides a detailed account of the IFIP intervention, including FPE and BFIS. BFIS includes offering patients at least one conversation where the major part is dedicated to discuss family involvement and FPE, offering relatives at least one conversation without the patient present, and inviting the patient and relative(s) for a conversation together. Structured conversation guidelines was developed to standardise the content of these conversations. BFIS also includes written information, crisis/coping plans and psychoeducative seminars for relatives. Further details of the planned intervention and implementation support can be found in our study protocol [30], and in a publication reporting fidelity outcomes (manuscript submitted).

Change in fidelity to the intervention (defined as the degree to which a program implementing an evidence-based practice adheres to specific model standards [32]), constitutes the IFIP trial’s primary outcome [30]. Statistical analyses of fidelity outcomes (the quality of the clinical interventions, penetrance, and organisational implementation, measured with three different fidelity-measures) show significant differences between experimental and control conditions (manuscript submitted). This sub-study aims to explore what factors inhibit and promote the implementation of family involvement in CMHC units, both in general and more specifically within the context of a large-scale implementation study. To our knowledge, this paper is the first to explore what actually facilitates the implementation of family involvement for persons with psychotic disorders in CMHCs, as part of a successful and large-scale implementation study. Furthermore, the IFIP implementation strategy is probably also unique, since it combines both basic and comprehensive family interventions.

The following research question guided this study: "What organisational and clinical barriers and facilitators do local implementation teams and clinicians in CMHCs experience when implementing family involvement for persons with psychotic disorders?” We also explored local variations within and between the participating units. In this study, barriers are defined in the following way: “Factors are considered as barriers if they impede implementation of, or adherence to the guideline”. We further define facilitators as follows: “Factors are considered as facilitators if their presence promotes the implementation of, or adherence to the guideline” [33].

## Methods

This article conforms to the *“*Standards for Reporting Qualitative Research (SRQR): 21-items checklist” [34] (Additional file 1).

### Study design and context

This study employed a qualitative approach, including both process and formative evaluation, nested within a cluster randomised implementation study [35]. Before the implementation period, we developed the IFIP intervention and drafted a summary of the most important barriers to and facilitators of implementing family involvement in mental health care. This work was based upon available guidelines, literature reviews, and extensive dialogue with the stakeholders. After inclusion and randomisation of the CMHC units, each clinical site in the experimental arm established a local implementation team (3–8 members) including dedicated clinicians and unit managers with a particular responsibility to oversee the implementation process. Throughout the implementation period, we explored how the IFIP intervention affected the stakeholders and the CMHC units through digital communication, face-to-face dialogue, ad-hoc meetings, planned teaching- and supervision activities, fidelity measurements, questionnaires, and qualitative interviews.

An important part of the IFIP intervention was the implementation support provided by the IFIP project group [3, 30]. One element of this support was a written summary of key barriers and facilitators. Inspired by a responsive evaluation approach [36] and as part of the formative evaluation [37], the summary of barriers and facilitators was shared with, used, and commented on several times by the stakeholders during the implementation period. During this process, the IFIP project group and the CMHCs (the implementation teams, clinicians, and leaders), regularly discussed and dealt with barriers to and promoters of family involvement. Thus, in this project the researchers and stakeholders (patients, families, mental health professionals, and health institutions) all contributed to the ongoing knowledge production. The close cooperation offered ample opportunities to explore barriers and facilitators. Preliminary findings from the qualitative interviews, together with field notes and informal feedback from the stakeholders, continuously assisted the implementation and research process, making it possible to adjust and improve the implementation support, including the summary of key barriers and facilitators.

The present study is based on data gathered through focus groups with the implementation teams in the beginning of the implementation period and after 10 months of implementation support, and with other clinical staff after 16 months of implementation support. However, the interviews, the interview guide, and the preunderstanding and interpretations of the researchers and the participants were inspired and influenced by the responsive and formative evaluation used before and during the implementation period, and the dynamic and co-produced summary of barriers and facilitators.

### The focus groups—participants and data collection

Twenty one semi-structured focus groups with local implementation teams were performed during the spring of 2019 (M2-3 of the 18-month implementation period), and the winter of 2020 (M9-10), and with other clinicians/staff in the fall of 2020 (M15-16). A total of 75 clinicians and members of implementation teams (mainly clinicians and unit managers) participated, of which 27 participated twice (See Table 1).

**Table 1:** Key characteristics of the participants in the qualitative study

	**STUDY SAMPLE**					
	Members of implementation teams Initial phase of intervention (N = 38, 8 focus groups)		Members of implementation teams Middle phase of intervention (N = 39, 8 focus groups)		Clinicians Late phase of intervention (N = 25, 5 focus groups)	
**CHARACTERISTIC**	**N**	**%**	**N**	**%**	**N**	**%**
**Sex**						
Male	6	16	5	13	5	20
Female	32	84	34	87	20	80
**Age in years**						
20–35	6	16	5	13	7	28
36–50	11	29	16	41	11	44
51–70	21	55	18	46	7	28
**Prof. background/role**						
Section/unit manager	6	16	5	13		
Physician	4	11	3	8	4	16
Psychologist	5	13	5	13	16	64
Psychiatric nurse	14	37	15	38	1	4
Other	9	24	11	28	4	16

As a natural consequence of the study design, we chose a purposive sampling strategy [38]. We wanted to explore the experiences of the implementation teams because they were particularly engaged in the implementation process. Clinicians with less commitment to the implementation work were interviewed to include less engaged and potentially more critical voices.

The data collection was performed at the CMHCs by five members of the IFIP project group (KMH, MR, RP, LH, and KSH). Each focus group was carried out by two researchers; one conducted the interview while the other assisted and took written notes. Before the start of each interview, we provided participants with information about the study and obtained written consent from all participants. Semi-structured interview guides (separate guides for each of the three interview sessions) containing a list of main topics and questions to be covered (Additional file 2), guided the interviews. We aimed at eliciting participants` thoughts, beliefs, and experiences with factors that would positively or negatively impact the implementation of family involvement. In both interview sessions with the implementation teams, the participants were initially encouraged to speak openly, before asked to comment more specifically on the summary of barriers and facilitators. In the interviews with other clinical staff, participants were encouraged to talk about a few, selected barriers that the process evaluation had revealed to be particularly demanding (for instance the duty of confidentiality). All interviews were audio recorded and lasted for 60–90 min. After each focus group, notes were summarised in a brief report to highlight important topics and to make data more accessible to the remaining research team. Audio-files, transcripts, and reports were immediately transferred to and stored in the University of Oslo’s secure database (In Norwegian: “Tjenester for Sensitive Data”–TSD). Project members transcribed the interviews verbatim.

### Analysis

**Fig. 1:**
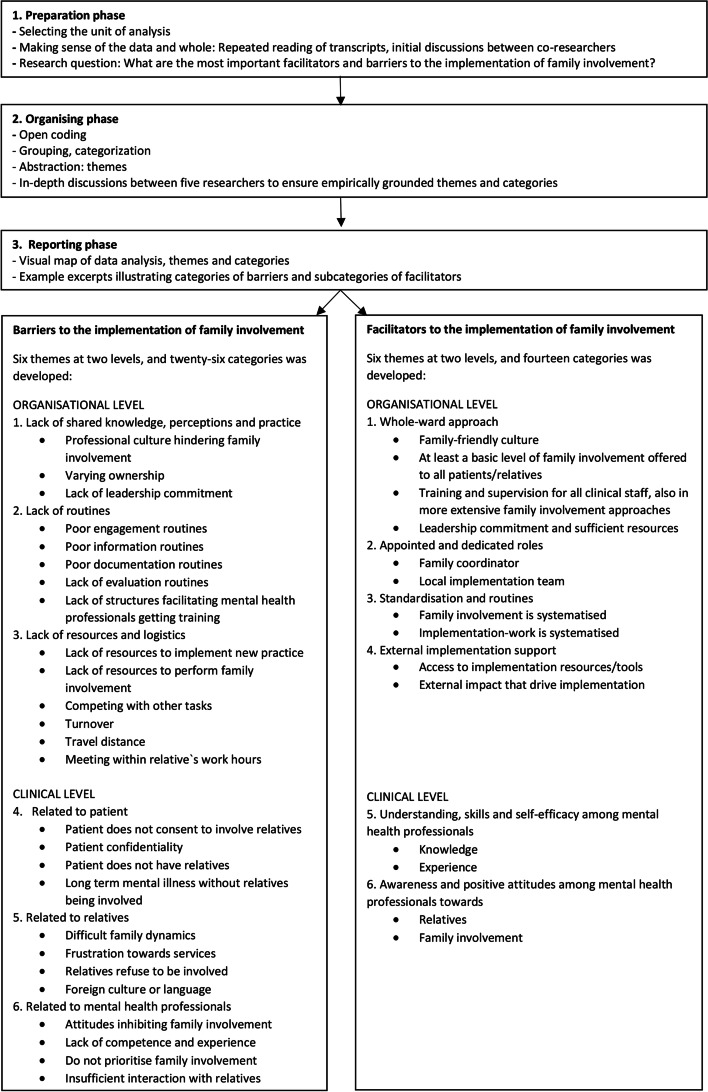
Visual map of data analysis with themes and categories

Analysis of the interview transcripts was carried out by the first author (KMH), using manifest content analysis according to Elo and Kyngäs [39]. Content analysis can be divided into three main phases [39]: the preparation phase, the organising phase, and the reporting phase. The preparation phase involved preliminary analysis of notes and brief reports from the first session of interviews with the implementation teams. This work further informed the development of the barrier- and facilitator document, which served as an implementation tool during the implementation period (formative evaluation). In addition, the preparation phase included thorough reading of transcripts (the unit of analysis) and brief reports in their entirety to achieve immersion and obtain an overview of the whole data. Furthermore, members of the research group (KSH, RP, MR and KMH) discussed preliminary themes regarding special topics of interest. The organising phase consisted of coding and categorising the material, guided by the research question. Initially, open coding of the text was conducted by labelling meaning units with initial codes, which were grouped into higher code groups and further collapsed into higher order categories. Structuring the data was done through an inductive approach; that is the categories were derived from the data, moving from the specific to the general [39]. This paper focuses mainly on factors that potentially had a positive impact on the implementation process. However, since we understand barriers and facilitators as highly intertwined, we performed the analysis with regard to both. The final abstraction process resulted in categories being grouped into six barrier- and six facilitator themes, before the material was scanned over again to ensure that relevant contents were placed in the right categories. Codes, subcategories, categories, and themes were adjusted, restructured, and renamed throughout the analysis process, continuously asking: “Why and how is this code/subcategory/category/theme a facilitating or hindering factor to the implementation of family involvement?” The NVivo computer software package 12 was used to assist with storage, searching, and coding of qualitative data. In the results section below, as well as in Fig. 1 and Additional file 3 and 4, the main findings are presented partly as condensed text [40] and partly as illustrative quotes. Quotes are presented in condensed form and in some places we have reproduced conversations between the researcher and the participant (P). We aimed to uncover the meaning content of the participants' statements, rather than bringing out all the details.

### Trustworthiness

To permit others to judge the quality of a study, one has an obligation to report sufficient details of data collection and analysis [41]. The reporting phase consisted of describing the step-by-step analysis (Fig. 1) and demonstrating defensible inferences from data to results [39] in coding schemes, including supporting excerpts (Additional file 3 and 4). Different types of triangulation [41] served as strategies to further reduce systematic bias and obtain trustworthiness. Members of the research team and an expert from The Early Intervention in Psychosis Advisory Unit for South East Norway (TIPS Sør-Øst) (KMH, KSH, MR, RP, BW and ISH) reviewed and discussed the way in which the data was labelled [42] and whether and how categories and themes were related to the research question (analyst triangulation). Data source triangulation was ensured by having mental health professionals with differing roles and perspectives participating in the focus groups, exploring what people said about the same phenomenon over time (comparing data at initial and late phase of the implementation). We also integrated ethnographic data on barriers and facilitators, derived through continuous feedback from stakeholders during the implementation period.

## Results

We identified six themes with a total of 26 categories representing barriers to implement family involvement: 1) Lack of shared knowledge, perceptions, and practice 2) Lack of routines 3) Lack of resources and logistics 4) Patient-related factors 5) Relative-related factors 6) Provider-related factors. The first three themes represent barriers at the organisational level, while the latter three represent barriers at the clinical level (Fig. 1).

Furthermore, we identified six themes with a total of 14 categories representing facilitators for implementing family involvement: 1) Whole-ward approach 2) Appointed and dedicated roles 3) Standardisation and routines 4) External implementation support 5) Understanding, skills, and self-efficacy among mental health professionals 6) Awareness and positive attitudes among mental health professionals. The first four themes represent facilitators at the organisational level, while the latter two represent facilitators at the clinical level (Fig. 1).

In the beginning of the project, when experience with implementation of family involvement was sparse, the focus was mostly on the barriers and more general or common experiences with implementation of family involvement. During the implementation period the experience with- and focus on facilitators gradually increased, as well as the more specific experiences with systematic implementation through participating in the IFIP trial. In previous research, barriers to the implementation of family involvement have been rather extensively explored, while knowledge about facilitators remains sparse [2]. Thus, in the present article, we focus on the facilitators. An overview of both barriers and facilitators is presented in Fig. 1, while Additional file 3 (barriers) and 4 (facilitators) provide additional illustrative quotes pertaining to the various themes, categories, and subcategories.

### Facilitators at the organisational level

#### Whole-ward approach

Prior to implementation, an important barrier was the lack of shared knowledge, perceptions, and practice. Family involvement practices appeared random, and seemed largely dependent on the individual professional’s interest and competence [3], hence at risk of falling apart.

In the IFIP trial, the clinical and organisational levels were approached simultaneously. An explicit aim was that all patients and relatives should be offered at least a basic level of family involvement and support. To enable this, all clinical staff were offered training and supervision, and most participated in the training. This may be described as the key elements of a “whole-ward approach”. The approach was experienced as consequential to develop a more family-friendly culture in most of the wards, as portrayed by this participant:

We take relatives into consideration in all settings, in all occasions, really. In the treatment team, reflective team, mini team. It`s hardly ever forgotten. There is something about our way of thinking that has changed. It is very evident with us. (FG12).

Many of the participants emphasised the importance of developing common understanding and priorities among the staff through the whole-ward approach, and enabling the staff to engage with the relatives in new ways:

It is an asset that so many of us have this training, because then we kind of have the same way of thinking about it. For example, that relatives to a greater extent are allowed to share their own experiences, that we are not just focused on obtaining information, or on the patient. (FG13).

Furthermore, the whole word approach was necessary for all clinicians and managers to acknowledge family involvement as a key ingredient in good care and treatment, and FPE as a recommended treatment option:

P: It was so important that all the professional groups were included in the FPE course (…) That helps it spread wider. For example, since I have received the training, I have a different view of the patients’ treatment options. (The researcher further asks whether the participant thinks that the other therapists are feeling the same way):

P: Yes, I think they feel the same. (FG12).

Furthermore, the basic level of family involvement and more comprehensive approach (FPE) appeared to be mutually reinforcing. For instance, the threshold to invite the patient and relatives to more comprehensive family involvement seemed to be lowered by establishing a basic level of family involvement as default approach. This approach also seemed to further a process with stepwise consent, where consent for family involvement was discussed several times and obtained gradually in an ongoing process, starting out with the most basic type of family involvement. This was considered better than an “all or nothing” approach. At the other hand, being trained in the more advanced model of family involvement (FPE) was perceived as useful when practicing basic levels of family involvement. Particularly valuable was the experience that the staff could utilise selected model elements, also when providing basic family involvement:

For instance, in conversations with patients and relatives I have used the FPE information material. To kind of make it easier to present it. So this has contributed to… I was about to say… to the regular conversations with patients and relatives in a positive way. (FG11).

A few participants mentioned that training all staff was time-consuming and was compounded by a high turnover of staff. Participants queried whether training would have been better with a small team of therapists working only with family involvement. However, in general the participants seemed to agree that the advantages of the whole-ward approach outweighed the disadvantages, particularly in a long-term perspective.

From a leader perspective, shared competence also contributed to strengthening the working environment and treatment practices because staff started to work in the same way:

Having a similar professional foundation affects the working environment (…) It ensures the quality of treatment because we think and work more synchronously. (FG9).

Participants reported that the whole-ward approach led to all therapists initiating family involvement with all their patients from an early stage in the illness trajectory:

Especially with new patients, the initial focus on family involvement is much more present, and… yes, we try to really look into both the referral and the patient’s chart, how things are. In addition, we have started to work on a checklist, to become even more conscious of that structure, so it is the same for all therapists, not coincidental, dependent on who is passionate about family involvement or not. It should be a somewhat standardised routine to invite relatives to conversations early in the trajectory, and provide appropriate information about why we think this is important. (FG2).

Some participants reported that over time the whole-ward approach led to the emergence of positive attitudes among clinicians with regard to engaging families. This was in contrast to involving families as a token gesture obligation:

Initially, it was kind of not so important, and it was just like “is it done or not”, but now it is on the checklists and markedly permeates the attitudes. A significant change has taken place, from family involvement representing an administrative measure towards being implemented in each individual persons practice. (FG12).

Leadership commitment, through practical adjustments and motivational support, played a pivotal role in the realisation of various facilitators identified in this study. Barriers to implementation—such as competing tasks, lack of resources, and varying ownership—were surmounted by leaders who allocated sufficient resources, appointed dedicated positions (see below), and used the IFIP intervention in a standardised way. Leaders that held a long-term perspective and identified a clear change in team practice as family involvement was prioritised and valued, were vital to the implementation. Important examples include making a working plan that allowed all staff to participate in training and supervision, and to run FPE-groups, stating clearly that offering family involvement is mandatory and that FPE-sessions allowed for a reduction in other therapeutic sessions. Some leaders also mentioned another way to limit the resources spent by the CMHCs; to run FPE-groups in collaboration with municipal health- and care services. In Norway, the CMHCs are by and large part of the specialised health care services (together with the hospitals), so this was also mentioned as a way to improve coordinated care and collaboration between specialised health care and primary health care services before discharge or transfer.

#### Appointed and dedicated roles

To guide the local implementation effort and operate as a link between the unit and the IFIP project group, each unit in the intervention arm was recommended to appoint a local implementation team and a family coordinator. Overall, study participants experienced that having such dedicated positions was a key facilitator. Especially two main tasks conducted by the local teams and the family coordinator were reported to strengthen implementation: Organising the various interventions, for example by preparing, establishing, and disseminating routines for basic family involvement and FPE at the unit, and to keep staff motivated and committed when faced with stressful workdays and competing tasks, for example through involving all the staff in the development of locally adapted routines.

Not all the family coordinators were allocated time for the intended tasks, and there was some variation in which tasks the various family coordinators performed. However, most coordinators played a fundamental role during the start-up phase by promoting awareness of the implementation, “keeping the family involvement warm” and by contributing to the overall competence development in family involvement. The coordinators provided training and supervision to their colleagues, and several participants pointed out the low threshold for obtaining their help and guidance with challenging cases:

I find that our two coordinators are core resources in reminding us of relatives' rights, and how important they are. I think that we need them. My experience is that I can’t cover all bases as a therapist, so it is nice to have them on the team. They remind me of something that is natural to them, but has not always been to me as a therapist, having been used to mainly focusing on the individual patient. (FG17).

Concerning the implementation team, regular team meetings (often bimonthly or monthly) and working together as a team of enthusiastic personnel with the unit manager were factors reported to strengthen the implementation:

It requires very dedicated people (…) that kind of are passionate about working with relatives. This is crucial, and something that I notice in all quality development projects. If an implementation team does not have these very dedicated people, you are off to a poor start. (FG14).

Nevertheless, some of the implementation teams did not function optimally, with a lack of leadership commitment being one of the explanations:

I do feel that as a leader I haven’t done enough to schedule, invite, and prioritise the implementation team meetings. I have not taken that responsibility as I should have done. (FG11).

Some participants mentioned that varying commitment among staff hampered the implementation process. There appeared confusion as to how to share responsibility and implement tasks effectively at the unit. Finally, a few participants mentioned that extensive commitment by the implementation team led to other clinicians withdrawing from engaging in family involvement, thinking that family involvement was not their responsibility.

#### Standardisation and routines

Participants` accounts highlighted the need for organising both the family involvement practices and the underlying implementation work systematically. At baseline of the IFIP-trial [3], most of the units’ family involvement practices suffered from a lack of standardisation with poor engagement, information, documentation-, and evaluation routines. Systematising family involvement, for instance through written procedures and information leaflets, documentation templates, and systems for routinely developing crisis plans and inviting all relatives to relevant evening seminars/courses, reportedly promoted implementation.

Standardisation also reportedly promoted normalisation and anchoring of family involvement as an integrated practice among all staff. Particularly during the start-up phase, the establishment of routines, procedures, and checklists was considered very important to ensure that family involvement was actually performed:

When I worked in somatic health care, we were supposed to call the relatives within 24 h. I think it is natural that we do the same thing here, just call within a day or two to hear how they are doing and if they have any questions. To me, I assume, this is where the shoe pinches (…) If it’s in the procedures, you just do it automatically, right. There is no need to wonder, that’s just the way it is (…) Then we have established contact with the relatives and can catch things at an earlier stage (…) It would have been very helpful if we had a procedure assigning the responsibility to make a call to the relatives, to one of staff. (FG1).

Some participants emphasised standardisation of patient conversations about family involvement as a means to better engage with the most severely and chronically ill patients. Many of these patients hadn’t previously engaged family in their care, which led to a break down in close relationships, sometimes permanently:

It will be good to concentrate more on offering all patients a conversation about family involvement. Because I believe that ensures that we’ll ask, even when the patient hasn’t involved his family before. That the therapists do not just assume that the patients do not want it. (FG11).

Some highlighted the importance of «flexible standardisation» and tailoring family involvement to the needs of each individual patient and family, such as this clinician describing how some relatives are more experienced in the role than others, thus having different needs:

…to establish contact, that applies to most of the patients, and is effective (…) while several other factors are more individual. It`s not always like "the more, the better". Because, you also have relatives who know a lot, and already have a lot of information. They may need something other than those who are relatives to a patient who has recently been diagnosed with a psychotic disorder. (FG17).

Some participants described feelings of fatigue due to an overload of checklists and procedures. To meet such challenges, one of the units successfully introduced adjustments to fit the intervention to the local working culture and level of competence. They decided to establish a “procedure for family involvement” which all clinicians were encouraged to follow, but without having to tick off that the tasks were done, as in a checklist:

We are absolutely allergic to even more checklists where we have to tick off whether we have done it right. We cannot stand it. But (we want) a list of ideas for how to proceed and what is prudent to do (…) Not mandatory, but more as a support. Designed for adult, responsible therapists who know that they should—and want to do their job. (FG13).

One advantage of such standardisation is that the procedure is available to all clinicians. Several months after the implementation, the manager at this particular unit reported that the procedure had been very useful when faced by staff turnover.

Another way to integrate family involvement was to secure that family involvement was always on the agenda in regular treatment meetings and included in all types of plans, e.g. work plans, treatment plans, capacity building plans, and discharge summaries. Furthermore, developing a clear plan on how to get started with FPE groups immediately after training seemed to be vital to get the most out of the FPE training and subsequent supervision, and also to increase the number of patients that were offered this kind of treatment. One way to achieve this was to have the family coordinator register patients and relatives who needed more comprehensive family involvement, and to match this list with available staff with FPE training. This could also be a way to prioritise FPE treatment fairly, if the units FPE capacity was not sufficient.

Some also mentioned the need to define required qualifications and a formal job description for the family coordinator:

Formalising the work… that the family coordinator holds an assigned position with competence requirements (…) is a way of making the family involvement visible. To me that would signalised that one took it seriously. (FG12).

#### External implementation support

Access to implementation resources from the IFIP project such as fidelity monitoring, training in FPE, and ongoing external support and supervision was considered crucial. External support was reported to be particularly important in helping the units to get started, generate the imperative of family involvement, build enthusiasm, and promote the implementation:

The most important thing is that we got help to sit down and look at what we have…, those fidelity assessments sort of confirmed what we already knew… And the fact that we did not let go… Even in difficult times. Having an implementation team, trying to get started with the groups and systematising our practice, we had not achieved that if you were not there, on the field with us. Because it has driven us. So I'm very happy about that, because otherwise it would have faded out, I'm pretty sure. And the supervision is “gold”. The training and supervision provided by TIPS Sør-Øst has been very important (…) Fantastic, yes. (FG13).

Some participants expressed concerns regarding the sustainability of ensuing family involvement when the external support was no longer available:

I have had such negative experiences throughout the years (…) a lot is invested in various things, but when the follow-up disappears and management takes over… This is what I worry about the most. That shift. (FG12).

### Facilitators at the clinical level

#### Understanding, skills, and self-efficacy among mental health professionals

At baseline, participants reported that clinical staff were often unfamiliar with family involvement prior to training and experience. Several participants feared that involving relatives would jeopardise their therapeutical alliance with the patient. They lacked understanding of the significance of services involving and supporting the family, and they lacked sufficient knowledge and skills to conduct family involvement. Training in FPE reportedly promoted understanding of the significance of involving and supporting the family, and contributed to increased understanding, skills, and self-efficacy among participants:

The FPE education has made me more structured with regard to family… that is, I have received a method and confidence—and especially quantity training and practice (…) and I have received supervision along the way. Then you become more confident. (FG15).

Clinical practice with regards to introducing family involvement to the patient and establishing contact with the family was noted to improve in response to training and experience. Several participants described how lack of knowledge and uncertainty previously meant they refrained from involving relatives, especially if the patient was reluctant:

One of the first patients where I was supposed to do it… you know, call the relatives… then the patient said: “No, you are not allowed to do that”. So. Yes. That was it (laughing). (FG4).

During implementation, when participants increased their knowledge and self-confidence on how to approach patients and relatives, core barriers such as lack of consent and the duty of confidentiality were dealt with in constructive ways. Conversations with patients about family involvement performed by skilled personnel provided them with information about how they could benefit from involving their closest relatives:

I think that they (colleagues) have improved in kind of introducing family involvement to the patient. For instance, taking that course taught me how to present it. If the patient says no right away, we do not resign, but continue to raise the issue. (FG3).

With increased competence and experience, clinicians started to explore why the patient was reluctant, if this was the case. They also became more confident on how to tailor family involvement to the patient’s needs, and to deal with the situation to benefit the patient and the relatives, thus increasing the odds that family involvement would actually take place.

#### Awareness, attitudes, and motivation among mental health professionals

Throughout the project, particularly in the beginning, many participants described how barriers related to mental health professionals (such as negative attitudes, lack of awareness and prioritisation of family involvement), barriers related to patients (such as lack of patient consent, confidentiality issues, and patients suffering from long-term illness without relatives being involved), difficult family dynamics and relatives` frustration towards services (see Fig. 1), hindered family involvement. This clinician emphasises the value of getting in touch with the relatives at early stage to prevent the patient's social network from dissolving:

It is important to establish early contact to prevent burnout and exhaustion. If relatives do not feel like been taken care of early enough in the process, the likelihood of them discontinuing contact with the patient increases (…) (thus some patients) live in group homes in which their closest next of kin is the personnel who work there. (FG14).

Participants reflected on how the involvement in the training and practice led to an increased level of awareness and appreciation of the importance of family involvement as an important element of treatment:

I feel that family involvement is far more present now. It is discussed every Monday.., also the FPE groups, we discuss much more… I feel that the role clarification is much clearer, more staff are engaged and the coordinators have the main responsibility. We are more conscious about family involvement, all the time. Talk about, ask for, clarify… relatives. And that is very good. (FG18).

Several participants noted that traditionally mental health services have neglected family involvement in the treatment of patients with psychotic disorders. Through the IFIP project, it became evident for many of the participants that this neglect, however widespread, is not very well justified:

So… I am a bit puzzled that we have been doing this for so many years without involving the relatives. It's a bit odd. (FG15).

With experience participants came to appreciate the benefit of the patient – relatives – therapists alliance. This also led to a greater awareness of the strain and challenges experienced by relatives and the importance of recognising and responding to career burden:

Just asking relatives a simple question like: "What is your experience as next of kin?” right. Just to get a question like that.., it's something that all relatives feel that they have never been asked. And when you are that vulnerable and exhausted… a large proportion are on sick leave due to the great burden of being a next of kin, imagine how valuable it is when someone asks that simple question! You don`t have to be a professional FPE-supervisor to manage that. (FG2).

Involving and supporting relatives at an early stage of the disease course also had an important function in preventing maladaptive interplay between patients, relatives, and health care personnel. Participants repeatedly identified the benefit of being trained in and practicing family involvement to help them understand their role and contribute to recovery:

P: We had this sick, sick girl. Then she moved to CITY (…) and the (therapists) there were much more committed to family involvement after they got that group (FPE) and saw the value in it (…) There were such ripple effects, I shudder when I say it (…) Basically (she was) very difficult to follow up or treat, but this group was the one thing that brought the family together. They thought that they (she) could not be released from HOSPITAL. But when they used problem solving techniques (core FPE-element to promote more adequate responses when symptomatic behaviors emerge) the moving process had gone well, so they were almost shocked. (Further the researcher asks what would have previously happened—and the participant responds as follows):

P: The parents would have been frustrated, wouldn’t understand and been angry at the treatment/clinic, at least that’s what happened before. Coercion, perhaps (…) inside a closed psychiatric ward. (The researcher then point out that these scenarios are quite different):

P: Yes (…) we need to think completely differently. Thinking of possibilities or… adapting to the individual, looking more broadly at the patients’ needs and the family, alternative solutions and not simply “that's how we do it, medicine and then out and finished” (FG5).

In response to positive clinical experiences, participants reportedly felt more motivated to continue providing family involvement:

A successful FPE course was raised several times. I believe that it kind of inspired the therapists to think that family involvement is important (…), at least after one such complete FPE course which was very successful. And that was one of the most ill patients. (FG19).

We also found that clinicians` positive perceptions of- and experiences with the FPE-model strengthened the implementation of family involvement in general. For example did they convey that the model being evidence-based, containing useful clinical tools and that one could utilise selected model elements also when performing basic levels of family involvement (see whole-ward approach), had a motivational effect:

The overall FPE mindset, not just the FPE groups, is a useful tool when meeting the relatives (…) also “outside” the model. (FG13).

## Discussion

We have explored what barriers and facilitators mental health professionals in CMHCs experienced when successfully implementing family involvement in mental health care for persons with psychotic disorders. We found that organisational measures such as a whole-ward approach, leadership commitment, dedicated roles, standardisation/routines, and external implementation support facilitated the implementation and seemed to improve the handling of core barriers. At the clinical level, training and practice promoted improved understandings, skills, and self-efficacy, besides increased awareness and positive attitudes among staff that reinforced implementation. In the following, we will discuss the most critical facilitators across the organisational and clinical levels.

### Whole-ward approach and leadership

The IFIP implementation strategy was a well-planned effort to make the units embrace family involvement comprehensively. Training all staff and implementing processes to provide all patients and relatives with at least a basic level of family involvement, gave rise to a more family-friendly culture and lowered the threshold to get started with family involvement and FPE. This approach seemed to promote the normalisation and integration of family involvement into daily clinical practice. In some units, a crucial change in "default mode" arose; while previously the act of involving relatives required a justification, the new practice required a justification when *not* involving the relatives. This is in line with previous studies reporting that a high level of trained staff facilitate implementation [27, 43].

However, factors affecting implementation are deeply intertwined and located at different levels [27]. For example, competent and motivated staff is not sufficient to succeed with implementation efforts, since quality improvement strategies focusing on individuals alone are seldom effective [44]. In this study for example, we found that the implementation of FPE was hampered when the clinicians were unable to practice FPE shortly after training. This is in line with previous studies reporting that although training was able to ensure good levels of competence within trainees, once they returned to their previous job roles, the implementation of new skills diminished or disappeared [45]. Therefore, organisational commitment and strong leadership that facilitate appropriate timing of training and practice is of the essence when implementing FPE.

Various studies demonstrate that a lack of protected time and heavy caseloads are core implementation barriers [46, 47], something the IFIP participants also reported. Nevertheless, our findings indicate that the whole-ward approach may have contributed to resolve resource-related barriers. Consistent with previous research we identified that implementation participants are not passive recipients of innovations [48] and their behavior is strongly affected by peer group influences and the culture of the organisation [44, 49]. It is possible that characteristics of individuals and the implementation climate have an even greater impact on implementation than increased resources. For instance, the top-down recognition that family involvement was obligatory allowed clinicians to prioritise allocating time to relatives [50]. Some clinicians also experienced time savings due to reduced ad hoc contact with relatives and improved treatment, for instance relatives contributing to medication adherence, more rapid discharge, and preventing relapse.

Systematically involving relatives at the onset of illness also promoted implementation by preventing negative interactions, often characterised by distrust, uncertainty, poor communication, and withdrawal among patients, relatives, and professionals [51–54]. Professionals neglecting relatives [55] can potentially harm the triadic relation in the form of barriers arising, while approaching relatives in attentive ways can lead to positive interactions (Fig. 1, clinical level).

### Flexible standardisation

Standardisation, with some flexibility and room for local adjustments, promoted implementation. By implementing procedures, conversation guides, and treatment plans, the participating units provided directions for practice, prevented family involvement from being seen as “nobody's responsibility” [56], and ensured that family involvement took place. Implementing a standardised family intervention (FPE) also benefitted the implementation of a basic level of family involvement (see below). Nevertheless, as stated by Selick et al. [22] family involvement is not a "one-size-fits-all" practice, hence it is imperative to offer diverse family services and to elicit user preferences [57]. While initiating family involvement with all patients as a standard procedure, one should also make adjustments to patients` and families` varying needs, and standardised interventions should allow for flexible usage.

Too strict requirements for practice might provoke resistance and frustration among professionals. The IFIP study aimed to sustain clinicians` professional autonomy by welcoming local variations in how to set up the implementation. When successful, this further promoted acceptance and positive attitudes towards the intervention among participants, instead of potentially harming implementation through the rise of resistance and frustration. Family involvement is most likely to succeed in units that manage to tailor family involvement to each treatment course and that manage to balance clinicians` need for professional autonomy with imposed implementation tasks.

### Basic and comprehensive levels of family involvement are mutually reinforcing

Implementing a spectrum of family interventions, from basic to advanced, reinforced implementation*. *When initial contact with the relatives was established as a default approach, the threshold to invite the patient and relatives to more comprehensive family involvement (FPE) seemed lowered. This approach enabled a stepwise consent, which worked better than an “all or nothing” approach. The efficiency also of less comprehensive models is supported by a recent systematic review [7].

Training the staff in FPE facilitated the units` basic family involvement. Increased competence and recognition among staff, besides access to FPE model elements, increased accessibility [18] and laid the foundation for basic high-quality conversations with patients and relatives. FPE is a complex and resource-intensive intervention. However, also using selected elements of the model was experienced as useful to several of the participating units, while basic conversations about family involvement seemed to resolve initial FPE barriers.

### Family involvement must be learned and experienced

One of the most important findings in this study is the fundamental need for adequate training and ongoing supervision of health professionals, so that they can offer family involvement [26]. Neither clinicians nor managers explicitly mentioned the lack of training and supervision as a barrier. It may be difficult to acknowledge a lack of competence if you have neither learned nor experienced what is missing. However, lack of training in family involvement practices constitutes a core implementation barrier [2]. In Norway, training in family involvement has generally been given little attention in the health education system and in the health services. An illustrative example is that none of the participating CMHCs had annual training in family involvement of their clinical personnel at baseline [3]. Strengthening the training in family involvement within basic and higher education for health professionals appears to be one of the most important areas of improvement for the future. Until then, it seems like the health services must provide this training, in anticipation of the health education programmes taking more responsibility. Hopefully, studies like the IFIP trial, indicating that it is in fact possible to increase the implementation of family involvement, may inspire necessary capacity building both within the health services and in health education.

Implementing family involvement in the context of severe mental illness is a complex intervention that confronts multiple barriers and complex ethical dilemmas [26]. In the initial phase, we experienced varying degrees of skepticism and resistance among participants, and the barriers were often considered unsurmountable. But in many cases, core barriers, such as the duty of confidentiality, decreased or dissolved when the clinicians started to practice family involvement after adequate training. Ensuring that clinicians gained experience with family involvement became—rather unexpectedly to the researchers—one of the most powerful facilitators throughout the implementation process. One possible explanation is that several of the identified barriers partly derive from insecurity with regard to relatives and family involvement practices that was alleviated when trained health professionals experienced family involvement in real-life settings. Furthermore, the whole-ward approach gave most staff new insights on the significance of family involvement, and made units less vulnerable to individual preferences and staff turnover.

### Access to know-how and expertise

The external implementation support had a formalising, competence-enhancing and motivational effect. The units benefited from substantial research- and clinical expertise within the fields of family interventions, ethics, law, health services, and implementation. They were given access to various resources such as training and supervision in FPE provided by TIPS Sør-Øst, evidence-based training and support provided by researchers at the Centre for Medical Ethics (UiO), as well as access to relevant external networks. This most likely increased the legitimacy of the interventions, as highly educated clinicians often have more confidence in evidence-based training and interventions. In this project, the external monitoring and evaluation, combined with systematic feedback, also seemed critical to identify areas for improvement, and to tailor and adjust the implementation process. As successfully adopted interventions typically include personal and ongoing contact between the intervention developer and adopters [53], IFIP researches frequently reminded and assisted the units in their efforts, and engaged in mutual collaborations with participants that reinforced practice and research. Overall, it seems like the external support contributed to reduce complexity, increase acceptability and reduce unit costs associated with implementation.

### Strengths and limitations

The current study finds its strength in how knowledge is developed, through continuous input and interpretation over time and in conjunction with stakeholders outside the research team. Process evaluation gave the opportunity to investigate different levels and stakeholders while the implementation proceeded. Formative evaluation made it possible to explore which measures actually worked well, thereafter adjusting accordingly. Responsive evaluation, which means that we turned into dialogue with the participants and all key stakeholders before and during implementation, strengthened the knowledge creation. Overall, this provided us with composite and robust data on multilevel facilitators from the perspectives of actors within mental health services. We might assume that this increases the likelihood that our implementation efforts are useful and sustainable in real-world settings [58]. The credibility of the findings is enhanced through the presentation of a rich amount of illustrating excerpts [41].

As a result of the nested study design, the facilitators described are probably to some extent molded by the planned intervention elements. The qualitative approach used in this study cannot demonstrate causality, generalisable findings, or outcomes for the patients and their relatives. We hope that other data from the IFIP study, such as fidelity outcomes and the perspectives of patients and relatives will help to further explore the impact of the facilitators described in this study.

### Implications

Our findings can inform future efforts to implement family involvement in mental health services. Implementation strategies should employ a whole-ward approach fostering shared understanding, attitudes, and goals. Leaders must signalise prioritisation, appoint dedicated roles, facilitate standardisation, allocate sufficient resources, and ensure that all clinicians get access to training, supervision, and practice. A basic level of family involvement and support should be the standard approach at hospital admission, followed by further individually tailored family involvement, which preferably leads to FPE. The current study is limited to family involvement in CMHCs for patients with psychotic disorders, but the findings are most likely transferable to the implementation of family involvement practices for other services and other patient groups.

We encourage researchers to explore facilitators also from the perspectives of patients and relatives, to employ quantitative studies to test the causal mechanisms hypothesised in this study, and to investigate whether and how our findings can be extrapolated to the treatment of other psychiatric disorders such as bipolar disorder, severe depression, and substance abuse. Future research should also investigate how much external support health services need to implement recommended practices that are not yet integrated in health education programmes. The significance of regional and national policies on family involvement—for example as expressed in health law, financial systems and basic education—should be further explored.

In line with two recently published systematic reviews on relapse prevention in schizophrenia [4, 7], we recommend that policy makers and clinicians give priority to family interventions such as FPE in resource allocation and treatment planning. Health educational institutions should incorporate basic training in family involvement to counteract professionals´ negative attitudes towards family involvement, and lack of competence and self-confidence when facing relatives of patients with psychotic disorders. For the future, one could argue that the whole-ward approach should be extended to a “whole health care and education approach” where good family care starts in the health educations, and is further embedded in the whole health- and care services. To achieve these goals, guidelines should be complemented with sufficient implementation resources and support.

## Conclusions

Implementing family involvement in mental health services for persons with psychotic disorders is possible through a whole-ward and multi-level approach, with organisational- and leadership commitment, and access to external implementation support. Our findings indicate that providing training in family psychoeducation to all staff, followed by clinicians getting experience with family involvement, may lower or dissolve core barriers. Together with routines to offer a basic level of family involvement to all patients as a default approach, these measures facilitate implementation and promote normalisation and integration of family involvement in treatment. As with other evidence-based treatment interventions for psychotic disorders, we must for the future expect entire units to hold a basic competence in family involvement. Training in family involvement should be incorporated in future health education programmes.

## Supplementary Information

**Additional file 1.** Standards for Reporting Qualitative Research (SRQR): 21-item checklist.

**Additional file 2.** Interview Guides.

**Additional file 3.** Example excerpts illustrating barrier categories.

**Additional file 4.** Example excerpts illustrating facilitator subcategories.

## Data Availability

The datasets used and/or analysed during the current study are available from the corresponding author on reasonable request.

## Abbreviations

FPE: Family psychoeducation
IFIP: Implementation of guidelines on family involvement for persons with psychotic disorders in community mental health centres
CMHC: Community mental health centre
BFIS: Basic family involvement and support
TSD: Tjenester for sensitive data
TIPS Sør-Øst: Early intervention in psychosis advisory unit for South East Norway

## References

[1] Dixon L, McFarlane WR, Lefley H, Lucksted A, Cohen M, Falloon I (2001). Evidence-based practices for services to families of people with psychiatric disabilities. Psychiatr Serv.

[2] Bucci S, Berry K, Barrowclough C, Haddock G (2016). Family interventions in psychosis: a review of the evidence and barriers to implementation. Aust Psychol.

[3] Hestmark L, Heiervang KS, Pedersen R, Hansson KM, Ruud T, Romøren M (2021). Family involvement practices for persons with psychotic disorders in community mental health centres – a cross-sectional fidelity-based study. BMC Psychiatry.

[4] Bighelli I, Rodolico A, García-Mieres H, Pitschel-Walz G, Hansen W-P, Schneider-Thoma J (2021). Psychosocial and psychological interventions for relapse prevention in schizophrenia: a systematic review and network meta-analysis. Lancet Psychiatry.

[5] Pharoah F, Mari J, Rathbone J, Wong W (2010). Family intervention for schizophrenia. Cochrane Database Syst Rev.

[6] Pitschel-Walz G, Leucht S, Bäuml J, Kissling W, Engel RR (2001). The effect of family interventions on relapse and rehospitalization in schizophrenia—A meta-analysis. Schizophr Bull.

[7] Rodolico A, Bighelli I, Avanzato C, Concerto C, Cutrufelli P, Mineo L (2022). Family interventions for relapse prevention in schizophrenia: a systematic review and network meta-analysis. Lancet Psychiatry.

[8] Yesufu-Udechuku A, Harrison B, Mayo-Wilson E, Young N, Woodhams P, Shiers D (2015). Interventions to improve the experience of caring for people with severe mental illness: systematic review and meta-analysis. Br J Psychiatry.

[9] Lobban F, Postlethwaite A, Glentworth D, Pinfold V, Wainwright L, Dunn G (2013). A systematic review of randomised controlled trials of interventions reporting outcomes for relatives of people with psychosis. Clin Psychol Rev.

[10] Ma CF, Chien WT, Bressington DT (2018). Family intervention for caregivers of people with recent-onset psychosis: A systematic review and meta-analysis. Early Interv Psychiatry.

[11] Helsedirektoratet. Nasjonal faglig retningslinje for utredning, behandling og oppfølging av personer med psykoselidelser. Oslo: Helsedirektoratet; 2013. Available from: https://www.helsedirektoratet.no/retningslinjer/psykoselidelser. Accessed Feb 2022.

[12] Helsedirektoratet. Veileder om pårørende i helse- og omsorgstjenesten. Oslo: Helsedirektoratet; 2017. Available from: https://www.helsedirektoratet.no/veiledere/parorendeveileder. Accessed Feb 2022.

[13] Helsedirektoratet. Psykoselidelser, inkludert mistanke om psykoseutvikling – barn, unge og voksne. Pakkeforløp 2018. Oslo: Helsedirektoratet; 2018. Available from: https://www.helsedirektoratet.no/pakkeforlop/psykoselidelser-inkludert-mistanke-om-psykoseutvikling-barn-unge-og-voksne. Accessed Feb 2022.

[14] Wallcraft J, Amering M, Freidin J, Davar B, Froggatt D, Jafri H (2011). Partnerships for better mental health worldwide: WPA recommendations on best practices in working with service users and family carers. World Psychiatry.

[15] The National Institute for Health and Care Excellence (NICE). Psychosis and schizophrenia in adults (CG178). London: National Institute for Health and Clinical Excellence; 2014 Report No.: CG178. Available from: https://www.nice.org.uk/guidance/cg178. Accessed Feb 2022.

[16] Galletly C, Castle D, Dark F, Humberstone V, Jablensky A, Killackey E (2016). Royal Australian and New Zealand college of psychiatrists clinical practice guidelines for the management of schizophrenia and related disorders. Aust N Z J Psychiatry.

[17] Westerlund A, Sundberg L, Nilsen P (2019). Implementation of implementation science knowledge: The research-practice gap paradox. Worldviews Evid Based Nurs.

[18] Lucksted A, McFarlane W, Downing D, Dixon L (2012). Recent developments in family psychoeducation as an evidence-based practice. J Marital Fam Ther.

[19] The American Psychiatric Association Practice guideline for the treatment of patients with schizophrenia; Third edition. Washington DC: The American Psychiatric Association; 2021. 10.1176/appi.books.9780890424841.

[20] Mueser KT, Deavers F, Penn DL, Cassisi JE (2013). Psychosocial treatments for schizophrenia. Annu Rev Clin Psychol.

[21] McFarlane WR (2016). Family interventions for schizophrenia and the psychoses: A review. Fam Process.

[22] Selick A, Durbin J, Vu N, O'Connor K, Volpe T, Lin E (2017). Barriers and facilitators to implementing family support and education in early psychosis intervention programmes: A systematic review. Early Interv Psychiatry.

[23] Szmukler G, Bloch S (1997). Family involvement in the care of people with psychoses: An ethical argument. Br J Psychiatry.

[24] Galletly C, Castle D, Dark F, Humberstone V, Jablensky A, Killackey E, et al. Royal Australian and new Zealand College of Psychiatrists clinical practice guidelines for the management of schizophrenia and related disorders. Aust N Z J Psychiatry. 2016;50(5):410–72. 10.1177/0004867416641195. 10.1177/0004867416641195 27106681

[25] Mairs H, Bradshaw T (2005). Implementing family intervention following training: what can the matter be?. J Psychiatr Ment Health Nurs.

[26] Landeweer E, Molewijk B, Hem MH, Pedersen R (2017). Worlds apart? A scoping review addressing different stakeholder perspectives on barriers to family involvement in the care for persons with severe mental illness. BMC Health Serv Res.

[27] Haddock G, Eisner E, Boone C, Davies G, Coogan C, Barrowclough C (2014). An investigation of the implementation of NICE-recommended CBT interventions for people with schizophrenia. J Ment Health.

[28] Eassom E, Giacco D, Dirik A, Priebe S (2014). Implementing family involvement in the treatment of patients with psychosis: a systematic review of facilitating and hindering factors. BMJ Open.

[29] Chen F (2008). A fine line to walk: Case managers' perspectives on sharing information with families. Qual Health Res.

[30] Hestmark L, Romoren M, Heiervang KS, Weimand B, Ruud T, Norvoll R (2020). Implementation of guidelines on family involvement for persons with psychotic disorders in community mental health centres (IFIP): protocol for a cluster randomised controlled trial. BMC Health Serv Res.

[31] Gilissen J, Pivodic L, Smets T, Gastmans C, Stichele RV, Deliens L (2017). Preconditions for successful advance care planning in nursing homes: A systematic review. Int J Nurs Stud.

[32] Bond GR, Evans L, Salyers MP, Williams J, Kim HW (2000). Measurement of fidelity in psychiatric rehabilitation. Ment Health Serv Res.

[33] Feyissa GT, Woldie M, Munn Z, Lockwood C (2019). Exploration of facilitators and barriers to the implementation of a guideline to reduce HIV-related stigma and discrimination in the Ethiopian healthcare settings: A descriptive qualitative study. PLoS ONE.

[34] O’Brien BC, Harris IB, Beckman TJ, Reed DA, Cook DA (2014). Standards for reporting qualitative research: A synthesis of recommendations. Acad Med.

[35] Stetler CB, Legro MW, Wallace CM, Bowman C, Guihan M, Hagedorn H (2006). The role of formative evaluation in implementation research and the QUERI experience. J Gen Intern Med.

[36] Abma TA (2006). The practice and politics of responsive evaluation. Am J Eval.

[37] UK Government. Evaluation in health and wellbeing. A guide to evaluation of health and wellbeing projects and programmes. Available from: https://www.gov.uk/government/collections/evaluation-in-health-and-wellbeing. Accessed Feb 2022.

[38] Palinkas LA, Horwitz SM, Green CA, Wisdom JP, Duan N, Hoagwood K (2015). Purposeful sampling for qualitative data collection and analysis in mixed method implementation research. Adm Policy Ment Health.

[39] Elo S, Kyngäs H (2008). The qualitative content analysis process. J Adv Nurse.

[40] Erlingsson C, Brysiewicz P (2017). A hands-on guide to doing content analysis. African J Emerg Med.

[41] Patton MQ (1999). Enhancing the quality and credibility of qualitative analysis. Health Serv Res.

[42] Graneheim UH, Lundman B (2004). Qualitative content analysis in nursing research: concepts, procedures and measures to achieve trustworthiness. Nurse Educ Today.

[43] Dirik A, Sandhu S, Giacco D, Barrett K, Bennison G, Collinson S (2017). Why involve families in acute mental healthcare? A collaborative conceptual review. BMJ Open.

[44] Ferlie EB, Shortell SM (2001). Improving the quality of health care in the United Kingdom and the United States: A framework for change. Milbank Q.

[45] Ince P, Haddock G, Tai S (2016). A systematic review of the implementation of recommended psychological interventions for schizophrenia: Rates, barriers, and improvement strategies. Psychol Psychother.

[46] Prytys M, Garety PA, Jolley S, Onwumere J, Craig T (2011). Implementing the NICE guideline for schizophrenia recommendations for psychological therapies: a qualitative analysis of the attitudes of CMHT staff. Clin Psychol Psychother.

[47] Cohen AN, Glynn SM, Hamilton AB, Young AS (2010). Implementation of a family intervention for individuals with schizophrenia. J Gen Intern Med.

[48] Greenhalgh T, Robert G, Macfarlane F, Bate P, Kyriakidou O (2004). Diffusion of innovations in service organizations: systematic review and recommendations. Milbank Q.

[49] Rowlands P (2004). The NICE schizophrenia guidelines: the challenge of implementation. Adv Psychiatr Treat.

[50] Chatzidamianos G, Lobban F, Jones S (2015). A qualitative analysis of relatives', health professionals' and service users' views on the involvement in care of relatives in bipolar disorder. BMC Psychiatry.

[51] Gray B, Robinson CA, Seddon D, Roberts A (2009). An emotive subject: insights from social, voluntary and healthcare professionals into the feelings of family carers for people with mental health problems. Health Soc Care Comm.

[52] Slade M, Pinfold V, Rapaport J, Bellringer S, Banerjee S, Kuipers E (2007). Best practice when service users do not consent to sharing information with carers. National multimethod study. Br J Psychiatry.

[53] Cohen AN, Glynn SM, Hamilton AB, Young AS (2010). Implementation of a family intervention for individuals with schizophrenia. J Gen Intern Med.

[54] Outram S, Harris G, Kelly B, Bylund CL, Cohen M, Landa Y (2015). ‘We didn’t have a clue’: Family caregivers’ experiences of the communication of a diagnosis of schizophrenia. Int J Soc Psychiatry.

[55] Kuipers E (2011). Cognitive behavioural therapy and family intervention for psychosis evidence-based but unavailable? The next steps. Psychoanal Psychother.

[56] Kuipers E, Yesufu-Udechuku A, Taylor C, Kendall T (2014). Management of psychosis and schizophrenia in adults: summary of updated NICE guidance. BMJ.

[57] Cohen AN, Drapalski AL, Glynn SM, Medoff D, Fang LJ, Dixon MB (2013). Preferences for family involvement in care among consumers with serious mental illness. Psychiatr Serv.

[58] Ramanadhan S, Davis MM, Armstrong R, Baquero B, Ko LK, Leng JC (2018). Participatory implementation science to increase the impact of evidence-based cancer prevention and control. Cancer Causes Control.

