# Supplementary material for: Barriers and facilitators when implementing family involvement for persons with psychotic disorders in community mental health centres – a nested qualitative study
Source: BMC Health Serv Res. 2022 Sep 12;22:1153. doi: 10.1186/s12913-022-08489-y (PMC9469513; doi:10.1186/s12913-022-08489-y)
Supplement: Supplementary file 2 — Additional file 2. Interview Guides. [file 12913_2022_8489_MOESM2_ESM.pdf]

IFIP study: Interview guide (initial phase) – Focus group interviews with local implementation teams

Overview of topics to be covered during the interviews

1. Experiences with the IFIP project so far

- What are the most important changes in the units' family involvement practices up until now?
- Experiences with the work in the local implementation team. What have you achieved so far? What works well / does not work?

2. Barriers and facilitators related to family involvement

- Now that you have begun working more systematically with family involvement practices:

a) Which barriers have you experienced to be the most important? What could be the reasons why only 0-5 % of patients with psychotic disorders receive family psychoeducation (FPE), and that the remaining receive little or no family involvement at all?

- On a clinical level
- On an organisational level and national level
- Any ethical dilemmas / conflicts of interest? (If not already covered)
- Are the barriers to family psychoeducation, and to family involvement practices in general, the same?

b) Which facilitators/success factors have you experienced to be the most important? What can be done to deal with/overcome the barriers? Which factors are critical to successfully increase the proportion who receive FPE, and to provide a minimum of conversations and information about family involvement to the remaining patients and their relatives?

- On a clinical level
- On an organisational level and national level
- Are the facilitators for family psychoeducation, and for family involvement practices in general, the same?

- Hand out the document on barriers and facilitators - does this correspond to what they have reported, and their experiences? Feel free to share specific examples.
- What could be the most important contributions from the clinical staff, the management, the local implementation team, and the project group? Is there anything that the relatives and the patients can do themselves?

3. Feedback on the implementation support programme/IFIP intervention

- Family psychoeducation
- Basic family involvement and support, in particular the conversations with patients, with relatives and the joint conversations for those who do not receive FPE.
- Other measures?
- Any suggestions for changes to the implementation support programme or IFIP intervention?

4. Any other experiences or views you would like to share with us?

IFIP study: Interview guide (late phase) – Focus group interviews with local implementation teams

Overview of topics to be covered during the interviews

Remember to ask for specific examples

Intro: Summarise the most important changes at the unit on all levels, check whether this information is correct, and then focus initially on the significance of the services that are increasingly offered to patients and relatives.

The significance of improved family involvement practices when in contact with patients and relatives (The clinical elements of the IFIP intervention: conversations, written information material, psychoeducative seminars for relatives, and family psychoeducation)

- For the patients.
- For the relatives.
- For yourself as health professionals, and the services.
- Is there anything else they should be offered?

Ethical dilemmas and conflicts of interest during family involvement, and other barriers and facilitators

- Which dilemmas/conflicts of interest have you experienced during family involvement, at the unit? (Patient vs. relatives. What roles do the clinicians' perceptions and interests play?)
- What challenges have you experienced concerning the exercise of the duty of confidentiality during family involvement?
- What challenges have you experienced with regard to receiving/documenting information from relatives?
- How were these situations handled? Could anything have been done otherwise?
- Hand out the 'barrier and facilitator' document and ask them to comment on any missing factors.
- Which measures could be useful at the administrative and policy level (health trust/national level)? (For instance legislation, financial incentives, documentation systems, more clearly stated policies on next of kin).

Experiences with the implementation effort (local implementation team, family coordinator, training, and guidance)

- The effort to implement the IFIP intervention. What works well or not so well at your unit? Possible changes? Any suggestions for other measures?
- Experiences with the implementation support programme? Positive and negative experiences? Any suggestions for changes?
- Is everybody in the unit committed to the project? Understanding of responsibility: What role and responsibility towards next of kin do you consider yourself to have, as health professionals? (Are there variations related to professional background?). Any changes?
- What impact do the clinical pathways for mental health and substance abuse have on the way you practice family involvement today?
- If we have time: Standardisation versus professional autonomy – what is a good balance?

IFIP study: Interview guide - Focus group interviews with clinical staff

Overview of topics to be covered during the interviews

Remember to ask for specific examples

Introduction: What are the most important changes that have taken place at your unit since the project began?

- How do you notice these changes in your daily work?
- Increased competence/assurance?
- Altered ways of thinking/attitudes?
- Altered ways of working?
- Changes in the services offered to the unit's patients and their relatives?

The significance of improved family involvement practices when in contact with patients and relatives (The clinical elements of the IFIP intervention: Conversations, family psychoeducation, crisis/coping plan, written information material, psychoeducative seminars for relatives). Positive and negative experiences. Ask a general open-ended question first, and then it is possible to ask specifically about each single element.

- For yourself as health professionals.
- For the health services.
- For patients and relatives.
- Specific examples.
- Any feedback from patients and relatives?
- Possibly mention the most important documented effects of family interventions and inquire whether they have experienced these effects.
- What has worked well at their unit, and why.
- What has not worked well at their unit, and why. Ask specifically about any suggestions for changes.
- Is there anything else that patients and relatives should be offered?

Challenges related to the duty of confidentiality and documentation

- Quite a few health professionals report that they face challenges related to the duty of confidentiality during family involvement. Have you experienced such challenges? Any changes?
- During the IFIP project, we have experienced that many clinicians are unsure of where and how they should receive/document information from relatives. Have you experienced such uncertainty? Any changes?
- (*If they report challenges*) How were these situations handled? Could anything have been done otherwise?
