# Supplementary material for: Barriers and facilitators when implementing family involvement for persons with psychotic disorders in community mental health centres – a nested qualitative study
Source: BMC Health Serv Res. 2022 Sep 12;22:1153. doi: 10.1186/s12913-022-08489-y (PMC9469513; doi:10.1186/s12913-022-08489-y)
Supplement: Supplementary file 3 — Additional file 3. Example excerpts illustrating barrier categories. [file 12913_2022_8489_MOESM3_ESM.pdf]

Example excerpts illustrating barrier categories

Theme/Category	Example excerpt(s)
**ORGANISATIONAL LEVEL**	
**1. Lack of shared knowledge, perceptions and practice**	
Professional culture hindering family involvement	Traditionally, in the health care trusts, the focus is primarily on the individual, and ... it is noticeable (...) that there is more resistance in team meetings for example, if you are to raise an issue in relation to relatives, then it turns out a bit like ... "Ok, but do we have to spend time on this? I am used to concentrating on the individual patient, so this is such an unknown area to me, so ... I do not want to get into it". (...) Well,… various attitudes and ways of thinking kind of clash.
Varying ownership	To get people to want to do that planning and organising (to find time to practice family involvement), they have to be dedicated, and far from everyone is. Thus, many of the statements uttered in this room are characterised by the fact that these three are particularly motivated. If you had asked some of the other FACT-team members, you would have gotten completely different answers, right (...) That is how it is with all quality development projects, right, you have those who are the most dedicated that engage in these implementation teams, and then you have others who are moderately interested , and then there are some who are sort of completely unengaged.
Lack of leadership commitment	There is a lot of fear [among clinicians] due to the possibility of lawsuits, right. Especially psychologists (…), if you breach confidentiality, you can be sued, and, (.. .) lacking support from the management, (...) there are huge, tough sanctions if you breach confidentiality (...) They have their reasons for being worried about it (.. .) I have seen some examples of that.
**2. Lack of routines**	
Poor engagement routines	My experience is that family involvement is much more implemented in interdisciplinary substance abuse treatment, (...) and procedures are much more established (...) When I used to work at an outpatient clinic, inviting the relatives was always part of a set structure, preferably in the first conversation to ... receive information.
Poor information routines	We' ve talked about two things that could have improved it, but were rejected: that the notification letters that are sent to new patients should involve something about family (…) or relatives; “Feel free to bring your relatives” (…) right, “here we work systematically with”…,, because the letters that are sent out, they are so long… terrible letters with all the rights you have in the health care system and in Norway and all kinds of information. Thick letter ... They do not read it, you know. They do not read it. What matters is what the top four lines say, with the meeting time and with whom. That’s what they notice, and then they show up with that pile (of papers) (…) In meetings we have raised…, NAME, the psychologist in the team, raised this issue; “Should this not have been changed?” And we just got these “No, it's impossible”-answers, from the administration, who said that “This should be the same for the whole hospital and lalala, like that. (…) We also raised the issue of the hospital website (…) I haven`t looked at it lately, but our website has never been particularly good. Or informative or ... So for the future… I know some hospitals have managed to make a website that says “We work systematically with family involvement”, encouraging, really.. In a more modern style, more information on the internet and in the notification letters so that family involvement was presented as an obvious, integral part of our job. We have proposed this, but so far it’s somehow decided on a level we do not control.

Poor documentation routines	One example from yesterday, actually (…), I was supposed to code a phone call with a relative. But there was no applicable code on the phone, it was, every other… (..) municipality, eeh, yes, every field but relatives had their own code (…) So we gave feedback about that to the mercantile staff, who could bring it up higher in the system, that they also want to make it (telephone conversations with relatives) visible in the coding. It`s very strange that it does not say. Hence, we have to code (relative) phone calls as patient calls (…) and that's not legitimate. I do not think that's right (…) you can imagine, when geographical distances are as long as here, a phone call can be very important sometimes, right.
Lack of evaluation routines	I feel that it is not just the information to the relatives that is insufficient, it is structures in general. Right. Such as, we say that relatives are important, but we do not evaluate what relatives think, right... I feel that there is a lack of structure with regard to documentation, evaluation, organisation, on many levels.
Lack of structures facilitating mental health professionals getting training	Something that was a bit unfortunate is that we got that package (FPE course) and everyone was into it/excited about it, but then we came back (to the unit) and everything we had put on hold was there, sort of. And then there were days off and it was holidays It would have been so nice if we had the chance to actually get started, right away
**3. Lack of resources and logistics**	
Lack of resources to implement new practice	It requires more from us, in fact, than medication (…) providing medication, that's much easier, right (…) If you are going to learn a new method, such as psychoeducation, that requires.., for a new method to "settle", then you have to learn to work in a completely new way, you,.. it takes a lot more effort. And since there is such a strong focus on productivity... and quality development is not something that you get money for, right, it will always be downgraded in a system that has such clear financial incentives. In overall that makes quality development remarkably challenging in our hospital. It is very difficult to achieve any quality development within this framework, it involves leaders, dedicated individuals and it requires someone who is willing to give a little extra. Because there is no room for it within the existing framework.
Lack of resources to perform family involvement	I probably think that the overriding challenge in offering good family involvement is resources. That`s a bit of a boring thing to say [unclear] but just to illustrate: If you are going to run FPE-groups, which I think is very useful and which I think a lot of patients will be motivated to do, then you need two therapists who have the FPE-competence in each and every conversation. Now I have one (therapist) in my team, further we have two therapists that are quitting, thus there`s just one left that knows how to do FPE. So, for every (patient) I'm recruiting, she must have one hour available (…). FPE sort of adds an extra dimension to the patient treatment. But, I also think more resources are required to do it.
Competing with other tasks	The framework with regard to the reorganisation, and DIPS arena (patient record system) Well… there`s a lot.. less people in the teams, it creates a lot more pressure because we have patients on compulsory treatment, right.. paragraphs and heavy medication, and those are things that we just have to prioritise. And the work week is no more than 37 and a half hours to 40 hours, so you have some very hard prioritising, I would say. That`s the main barrier I think. Because I believe people want to.., or I can actually only speak for myself, but I want to and I see that .. I believe that the patients benefit from it. Both during the conversations, but also as part of a positive long run treatment effect. But (…) it`s the timing, simply said…
Turnover	I do not know if it would have been beneficial to have a small group, two or four, working only with that (FPE). Because people come and go all the time, requiring constant training of new (group leaders)… groups sort of dissolve.
Travel distance	So far, sufficient financial incentives with regard to family involvement are not implemented (…) And when you do not get that, it is difficult for managers to get motivated for it, because they get no gain. It may sound a bit cynical, but ..., but that`s how they

	have chosen to administer our hospital, then, so that I ... eeh ... I, I ... hope it does not take too long before the financial gains are set, so that you get paid for talking to relatives. Consider this FACT team, right.., we are talking long travel distances. It can be a matter of driving one hour to a patient, and if you are going to drive one hour to talk to a relative, which is in line with all the main guidelines, and then you must return, then you are up to three working hours, that is, half a work day. Eh ... And, and if you don`t get paid for that, you can bet on a salary in heaven. But, our hospital isn`t organised that way, investing in salaries in heaven… you invest in salaries here…, the system is very cynical, though, financially controlled.
Meeting within relative`s work hours	One of the barriers is also that we must do it within work hours because, uh, those who are not patients [relatives] may have a job ... that makes it difficult to get here. But ... we will make it. I believe that having a hectic everyday life is a barrier for many.
**CLINICAL LEVEL**	
**4. Related to patient**	
Patient does not consent to involve the relatives	I think the greatest challenges with regard to psychoeducation, is to get the relatives involved. To get a consent from the patient to provide their relatives with some insight. Because the patient spends a lot of time keeping facades, keeping people at a distance, and in being healthy in their eyes, the eyes of the relatives. Thus, to them, it is completely out of the question to involve the husband or wife so that they get to know. "If they knew..," well, they are telling me: "If you knew how much energy I use to stay healthy. And be normal…». But here, in the therapeutic room, they are allowed some reprieve [unclear], to be who they are. But, then again they straighten up and walk out from that room. It is completely out of the question for them to involve their relatives. It is a big challenge.
Patient confidentiality	We try to be some kind of catalyst and to do a bit of "cleaning up" in their environment, but it is difficult (…) we are forced into an "in-between"-position where we try to take care of the patient, meanwhile the relatives are crying for any kind of hope of improvement, stabilisation, explanations on what is going on. To some degree we can give them that, but even when it is written in the F5 chart (part of the electronic patient record) that the relatives are listed as relatives, if the patient says "you are not allowed to call my father", suddenly they`ve changed it, right… And the patient's rights are so strong, and we must respect that. So that is certainly challenging.
Patient does not have relatives	..and besides, some do not have relatives. They live alone and their parents live far away and ... Well, they [the patients] are maybe in their fifties, sixties themselves. With old parents.
Long term mental illness without relatives being involved	P: I believe that these patients which have received family involvement since they were young and were transferred to RE (rehabilitation team),.. it is completely different than those who have been in the system for twenty years. I'm sure they [those receiving family involvement since they were young] are used to having their parents involved in a completely different way.<br>R: So you think it would be easier to continue a good collaboration if you had started earlier?<br>P: I think so.
**5. Related to relatives**	
Difficult family dynamics	Then, there are some families where it is, you know.., there are many kinds of families. Obviously, we make adjustments to what the family looks like. Nor should all relatives be involved. Unfortunately. But I assume those are the exceptions.
Frustration towards services	... and when you are under enormous time pressure, like we are, the production demands are heavy, you constantly have to produce, then when you get those, those phone calls, with so much anger and frustration, that ... it does not increase the motivation to work with relatives. You have to break that cycle, right, that`s my concern.

Relatives refuse to be involved	P1: There will be a meeting on Wednesday in the outpatient clinic that I will attend along with the chief physician, in relation to a family which has been invited in, where they do not want to have contact with the patient (…)<br>P2: Yes, but the relatives want contact with the services or with us?<br>P1: No, do not want, no<br>P2: Neither with us, nor the patient?<br>P1: No. The relatives do not want to be involved, but the chief physician invited them anyway.
Foreign culture or language	Non-ethnic Norwegians, obviously that is also a challenge. It can be both cultural, you know, in understandings and such, and it can also be linguistic (…) but often there are both linguistic and cultural barriers. It can be sort of difficult to manage. I believe we have something to strive towards here
**6. Related to mental health professionals**	
Attitudes inhibiting family involvement	In the long run, family involvement is an appropriate measure. Well, it has somewhat surprised me that have we kept on for so many years without involving the relatives more than we did, it is a bit weird. It does not hold a high status in the world of therapists. It`s about to change, but so far it has not been. It`s not what the famous therapists are doing (…) especially psychologists, what psychologists intend to do, they want to do cognitive therapy or (…) assessments. Doing family work is basic work I assume that many have been thinking "others should do, not us".
Lack of competence and experience	Many patients are considered not to have the capacity to consent (…) and the relatives often become part of the deficiency (…) or,.. yes, but true. And the relatives have immense needs (…) I think many of us find this so difficult to probe into that we avoid it, leading to limited contact with the relatives. And that is what we must try to change, right.
Do not prioritise family involvement	If we are going to think a bit broader. That this [family involvement] should somehow become an integral part of mental health care, I assume that, that it must be formalised and in one way or another, become as much a matter of course as very many of the other things we do (…) For example that to have offered proper - or good enough - treatment, family involvement is just as important as finances or counting, or routines. Because now, unfortunately, this [family involvement] is put on top of all the other things that we absolutely must do, therefore, unfortunately harder to prioritise. .
Insufficient interaction with relatives	I think we have an idea of…, I think that if we ask many of the relatives we are talking about, I am absolutely sure we feel that we give them more than they find that they get.
