# Supplementary material for: Barriers and facilitators when implementing family involvement for persons with psychotic disorders in community mental health centres – a nested qualitative study
Source: BMC Health Serv Res. 2022 Sep 12;22:1153. doi: 10.1186/s12913-022-08489-y (PMC9469513; doi:10.1186/s12913-022-08489-y)
Supplement: Supplementary file 4 — Additional file 4. Example excerpts illustrating facilitator subcategories. [file 12913_2022_8489_MOESM4_ESM.pdf]

Example excerpts illustrating facilitator subcategories

Theme/Category	Subcategory with example excerpt(s)
**ORGANISATIONAL LEVEL**	
**1. Whole-ward approach**	
Family-friendly culture	**All staff involved**<br>(NAME) somehow has a main responsibility, is the one having the main overview and does the most, but we are all involved and pull that load.<br><br>**Change of attitudes from avoidance to accept**<br>The project has also contributed to more openness. Daring to work with relatives. It is not that scary, it is not like... criminal. Like one used to think before. It joins the ranks of things that have changed quite a bit in a few years<br><br>**Staff increasingly recognising family involvement as appropriate treatment**<br>We experienced it most recently today with a patient. When we are stuck, which is not rare, however, you are stuck with "what do you do"? This is such a chronic issue. I recognise that family involvement and conversations with relatives, psychoeducation with relatives and perhaps this kind of structured FPE work, is actually a treatment method.<br><br>**Shared understandings and focus**<br>There are many benefits to so many of us having taken that course (FPE). You have that understanding and that focus with you, even though you may not actively work with groups.
At least a basic level of family involvement offered to all patients/relatives	**Establishing an alliance with the patient early in illness trajectory**<br>I can imagine that maybe sometimes, at least for the patient, the fact that family involvement is systematised, makes it easier to say yes when it is embedded in the assessment package; "We have this and this and we also have conversations with relatives and together"…<br><br>**Establishing contact and alliance with the relatives early in illness trajectory**<br>I also find that I just have to make a phone call to establish that initial contact, showing openness and interest in how the relatives are experiencing the situation. This can have a positive effect in that I get an alliance also with the relatives, not just with the patient.
Training and supervision for all clinical staff, also in more extensive family involvement approaches	**Utilising FPE elements in the basic family involvement**<br>If I do not have time to do the whole FPE from A to Z, I will at least try to use some elements that can be adjusted to the patient. (In that situation) I could not complete the whole FPE but at least I had two or three conversations with the relatives (...) And we had this other patient where we did not have time to talk to the relatives, but where we could use that problem-solving

technique from FPE. Without the relatives, but ok. That also worked well (...) To use and adapt the different techniques that we learned, and adjust it to the patient.

Prevents varying practice
We have observed that it varies within the (FACT) team to what extent they [the clincians] feel confident in working with relatives (...) But (NAME) told me not long ago that he perceives that those who have been least confident in working with relatives now to a greater extent can refer to conversations with relatives. Thus, perhaps the differences are about to be somewhat evened out.

Facilitates integration of family involvement
I think like that with all the things ([implementation] we have done this semester. We have done a lot of family work before, and we have had several dedicated individuals, right, who have worked with it, but it has never been sufficiently implemented as part of “this is what we do”. We got started on that now. We planned that we (should) arrange for one day with these TIPS people ([experts performing the training]) to brush up ... because many of us previously have participated in multi-family courses. But, the fact that all of us now were offered a four-day course, of course that was absolutely, uh, essential to get as far as we have today, already.

Facilitates that staff practice family involvement
Those who took that education (FPE) were fully aware that this also obliged them to run groups (…) You cannot just get that education and not being active. This is a great improvement from before, now it was a matter of course that if they took that education they should be running groups as well.

Utilising FPE-elements enables clincians` practice
It was very important for us to realise that we can use elements of the model . Because then you can practice the elements. You can do that more frequently than ... (…) because we don’t manage to start, eh ... enough groups at any given time to gain enough training, so that ... using FPE-elements is good way of practicing.

Customised supervision
When I was running groups I found that the supervision was absolutely essential (…) especially as a novice. When you've done it a lot, it's easier, but as a novice it was totally... eh.. yes, very helpful to have the opportunity to return with your issues. Then, it was important that the supervision was so frequent that you (…) or to that extent available, so that you could “handle it here and now”. We did not have continuously access to supervision but having the opportunity to call them ahead of the next session when you needed it ... I find that very useful. Because when we simply weren`t getting anywhere, it would be silly to run five more sessions, before you get that supervision…

All staff are offered FPE-supervision
The management were very concerned that those who should be allowed to participate in the FPE-supervision that should only be those who were running these standarised FPE-courses. But then it turned out that just three people or something like that,

	three, four people would end up participating ... But, now I have been, eeh, very determined, and made them accept that those supervision sessions should be integrated into more mandatory ... working hours, which is mandatory meeting time. FPE should be accessible to everyone", with the purpose of implementing some kind of FPE-light, as I call it.
Leadership commitment and sufficient resources	**Positive management attitudes**<br>Unit leader: It is about prioritising tasks, right. As an outpatient clinic, we have an overall budget and we have funding that we are required to deliver on (…) but it is a prioritisation to achieve a coherent and proper treatment process, right (…) Our health care system has become a place where we count what we can produce., like they do in, well, in real companies that produce things, right. And naturally, you ([the therapist]) can be left with a feeling…, what is in fact my contribution? So, when I have a conversation with an employee, for example, someone who mainly does IMR groups ([Illness Management Recovery]) (…), it's about contributing to the whole, right(…) helping people to understand what their role is, why it is important. Regardless of having a thousand codes, or you may have a hundred (…) running groups is more time consuming, right (…) it is about everyone carrying their essential role into the whole, so as many ([patients]) as possible are receiving the best possible treatment at our outpatient clinic.<br><br>**Appointing dedicated positions**<br>Family involvement should be formalised in a dedicated job position that is somehow visible. When someone quits, for example, others can apply for that position (…) I also believe that another way of making family involvement more visible is to make competence in family involvement a requirement in job advertisements.<br><br>**Facilitates family involvement are regularly tematised**<br>In addition, there is an increased awareness regarding setting aside time in meetings. That we have a structure for it, and this must necessarily be initiated by the management. Team meetings, morning meetings. Because ... eh, yes, my experience is that finding time is a challenge, everything is so important, so setting aside time for both the pediatric work and the adult relatives...<br><br>**Resource allocation**<br>We have experienced lots of those challenges here, they are well known. What set about the turnaround operation, was actually when family involvement was put into a system. And it was in a way equated with the other treatments. Resources were set aside for it. It was more acknowledged, simply put. .<br><br>**Signalises prioritising**<br>We also have an introductory course for all new employees. Mandatory, displaying to all new employees that family work is an expectation here.<br><br>**Ensuring staff get started with FPE-groups after training**<br>Many people have been admitted to that education here before, but some of them never get started (…) perhaps they are a little reluctant (…) Therefore it is important that… now they that have taken the course, the first thing is that they get started with groups while we will focus on the structure as we go.

	**Long term perspective**<br>What sold me on it when we were introduced to it ([the project/family involvement]) was that it is supposed to be offered as an integral part of the treatment, right. That it should not come in addition to it (…) We are continuously imposed with tasks that come on top of what we already do. It is seldom followed by someone saying: "To compensate we will reduce some others tasks, right..." It is always on top of it. So, as I said, there are some minor costs with regard to the staff but in the long run I think it will lead to a considerable boost, right, that you get it as part of your everyday work. That is also because now everyone has a greater understanding of what family involvement is and how they can benefit from it, right.<br><br>**Facilitating coordinated care across specialised- and primary health care**<br>Another positive thing related to FPE was that the municipality was involved in it and have more knowledge. It improves the collaboration (…) We have experienced it in (NAME municipality) right, where they are very conscious about FPE., where patients start with family involvement in the specialised health care services, and if they are transferred to the municipality, this is done in collaboration with employees in the municipality who have the same competence. This is the way to go further, to spread the competence. Because high turnover is a challenge in the specialist health service because we have many who are taking further education. So that we indeed will be experiencing turnover here. The municipal health services, however, have a much more stable group of employees. And the expertise is great. So it would have been very nice to establish FPE as a method of coordinated care, quite simply.
**2. Appointed and dedicated roles**	
Family coordinator	**Main responsibility**<br>The family coordinator in the psychosis team is responsible for follow up on the relatives. Because we have a family coordinator that has the main responsibility, it has been much more systematised, I think. Organised (…) that always… yes, remembers it and is nagging us about that stuff. So, that's good (…) There's a lot that should be remembered and done for each patient, (…). When someone has that function, it is not... then it gets done.<br><br>**Creates routines and structure**<br>I really believe in establishing a family coordinator role. It is of course related to knowledge that X mentioned, and structure, but having someone who is responsible for driving the family work, it is something that I see generally in all professional development projects… Because before I had the impression that family involvement was much more random, depending on the individual therapist, (whether he / she) had a personal interest in relatives or not that was decisive whether the individual therapist followed up the relatives or not. While now it is much more systematised, right, now there are standards on how to have conversations, in what way and yes…<br><br>**Provides training and guidance to colleagues**<br>Sometimes the closest relative…, what shall I say .., does not act in the patient's best interest. And then it is perhaps extra important that we bring in X ([the coordinator]), to deal with it. Because we find that some relatives act inappropriately, or have an unfortunate impact on the patient (laughs a little) (…) and the patient suffers from it. And then it is extra important to work on

	it, and it is very good to kind of have X to lean on, who can approach the case from a slightly different point of view than us. So that it is not just discussions about treatment.<br><br>**Initial dialogue with all patients**<br>My experience is that very few patients prohibit us from providing the relatives with that kind of information. But, that's because every (patient) is assigned a conversation with me where they get information about the family involvement that we offer. All new patients. And I'm the one who approaches (the relatives) and sometimes it's the therapists who do it. Besides, I believe that the therapists speak a lot with the relatives now. That`s my impression.
Local implementation team	**Dedicated personnel and unit manager in team**<br>R: Is there anything you want more of, or have not received that could be useful in this type of quality improvement project?<br>P1: It would have been useful to have the leader present in the (implementation) team.<br>P2: Exactly what I was going to say (several participants: yes, etc.)<br><br>**Regular team meetings**<br>It requires that you have time for preparation, you need time to somehow get mentally prepared, and.., and talk together afterwards and such, and there is not much time for that, you know. . So perhaps we have to get some help in a meeting to sort of, "ok, we have a patient who is in the target group, and then we should make a group, finding two therapists to be included in the group. This meeting on Fridays… that we can discuss family involvement, put aside some dedicated time together, I believe that... makes it easier to get started. If the four of us manage to raise this flag, then I think we can help each other to... get started.<br><br>**Promotes awareness, knowledge and motivation among team members**<br>R: Now that you`ve been working in this improvement team for a few months… Would you like to say a little about how it has been? Is it important to have someone who is especially dedicated to get this work going and, yes, how has it been to work that way, both for better or worse?<br>P: It has been very interesting and ... uh, yes, I have learned a lot. And... gained a much greater awareness of the importance of it. I believe that we succeed in collaborating and that inspires me and I see, see the impact it has.<br><br>**Practical work to facilitate family involvement measures**<br>So far, we have planned two teaching seminars for relatives that have been sent out, but they don`t make themselves. . There's a lot of work with the marketing, you can't just send something out…, you have to send physical invitations by mail, call around to the leaders of the municipalities to motivate them, get access to meetings so that the leaders can further motivate their therapists to inform the relatives. Thus, there is a lot of practical and somewhat invisible work to be done.<br><br>**Influencing the management**<br>Leader of implementation team: The two family coordinators that we have are great, but if they are not taken care of with proper structures, if they are not given dedicated time…, it's about negotiations, about working hours, working systematically with

	management, anchoring and creating structures that make the family involvement stick. It is (...) important, because the coordinators last for a project period so that you might get good evaluations of this particular project, but they do not last over time. Therefore, I am much more concerned with structures, structures that will last.<br><br>**Teamwork**<br>R: What has it been like, working within an implementation team?<br>P: It has been nice, but that, it`s a lot about the collegial dimension. That we work well together as colleagues. We plan groups together, we sit there with relatives, thus, good collaboration is important..
**3. Standardisation and routines**	
Family involvement is systematised	**Routines to support clinicians**<br>We need the family involvement to be more structured … there are a lot of different things to keep in mind. Now we have clinical pathways, where we have to remember the coding. Further, all treatment plans must be revised every third month, then contact with the relatives... There are a thousand things like that.., and we do not have a good system. I really need someone to help me with… reviewing, checking and all these things. If I`m going to do it all that myself... It takes a lot of time and you barely get through the regular daily routines. “Have you done it? Have you offered it? Documented it in the patient-chart?” All that stuff. .. Yes it easily gets pushed aside by all kinds of hubbub and other crises.<br><br>**Organising the engagement phase**<br>P1: Maybe we should make a routine that once a week we contact [the relatives]) by phone?<br>P2: This has to do with procedures.. Like who is going to do it…? Because the chief physician, she wants to control that part, so then it becomes a bit fuzzy- who is in fact contacting the relatives? If we managed to make a procedure that makes the primary contact responsible for doing it, then I believe it would get done. And perhaps a bit faster than today because when the chief physician wants the last word on who should call – whether we should call them at all, and at what time it should be done, it ends up being unclear.<br><br>**Flexible standardisation**<br>If the relatives know that the patient is here, confidentiality is not an issue. So we should have a system so that the relatives of patients who do not want them involved can get some guidance, too. Because they have the knowledge about these patients, about what they need. It`s not primarily us. So ensuring that they can get something without involving the therapist, I have found that to be incredibly effective. That`s how we do it (…) it is possible to solve it in other ways to prevent the relatives from just “floating at home alone” without getting advice, or any guidance. Be allowed to discuss with someone.
Implementation-work is systematised	**Ensure clinicians are allowed to practice**<br>I can spend day in and day out at courses, but practicing is the still most important. Mass training to ensure sufficient practice of the method is a necessity. I mean, it is such a classic problem, there is actually such a large turnover of staff here that people do not get enough experience. Being responsible for this for a long time now, I see it very clearly; it is not the case that when you

	have taken a course, you've got it. So, you have to practice and practice and practice (…) and have enough time to actually have enough groups. So that it does not get old (…) the longer time that has passed, the higher the threshold.<br><br>**Clear role distribution**<br>The nurses/social educators has the main responsibility for ensuring the family involvement. Not necessarily doing it, but to follow up that it gets done and.. that they have been contacted and stuff .. That makes it much easier for me. The roles are clarified.. Eh ..because we work in a slightly special way. The patient does not have one person responsible for him/her, several staff members are involved. Thus, it's good to know who does what, so we don`t overlap, or forget it.<br><br>**Replacing individual sessions with FPE-sessions**<br>But it takes a lot of resources s when two therapists have to do it... In the long run (…) because now we do both; we have individual conversations and we have PEF, right. It is not like we define that «okay now we are going to do this as part of the treatment»… This IS treatment, so for some (patients) it is not necessary to have so many individual conversations during that period. At the same time. Because now, we do both. And I think that's very important to remember. Because the point is: it's the same group! I think this goes for all outpatient clinics; we are very good at individual therapy, and consider the groups as an add-on(…) But when running groups replaces individual treatment… Yes then you can reduce the number of individual sessions, because this is part of the treatment.<br><br>**Setting aside dedicated time for family involvement**<br>We are also looking at how we can set aside time in the appointment books specified for family work. (…) How to solve this is a topic at the unit.
**4. External implementation support**	
Access to implementation resources/tools	**Fidelity monitoring and evaluation tool**<br>You have access to some tools, for example the fidelity measurements; "What are you actually doing?", right, the interviews, the focus you have brought with you.<br><br>**Training in FPE and other competence developments**<br>We are very grateful for the project because it helps us to focus and getting so many group leaders trained at once was a boost.<br><br>**Ongoing support and supervision**<br>I very much agree with you about that phone... Having it available quickly…It does not have to take that much time. But that someone is there and that you know them a little so that you're willing to make the call.. I think you have been very good at making our uncertainty feel legitimate, that we aren`t always able to do things right.
External impact that drive implementation	**Getting started**<br>We got great help regarding the course, getting it started. It was a lot of work getting it off it`s feet, getting people to join. And really highlighting what we are going for, right?

	**Formalising effect**<br>We participated in that kind of project with the Norwegian Medical Association a few years ago, we did not get those results, we did not succeed in the follow-up and continuity. It`s the same with the X-project. So I believe that the support in this project has been absolutely crucial to (…) avoid practice relying on dedicated individuals and to help formalise the family involvement.<br><br>**External implementers pushing implementation**<br>The communication with THE RESEARCHER becomes an incentive, right? «How are you doing, can we talk», it gives a push to keep working. Because, we have done quality improvement work before, and it is, it is hard work. That`s my experience. And when we have done it before, it is hard to get the ward and colleagues on board, because everyone has so many important things in their own head. So, I find, that this is the most successful quality improvement work I have been part of, and we have had a few. In relation to relatives, we have tried things before regarding family work.<br><br>**Creates awareness of own practice**<br>To me, it helps just to sit here and reflect and discuss, to look at where we can improve. That helps me, at least. So it`s better if you set aside time, just to have you here asking these questions I find very helpful, because, well. It makes it so clear (...), so clear in a way.<br><br>**Facilitates administration and structure**<br>It`s a while ago now, but you would hear «oh, another project». In a way, it felt, in the beginning maybe like a mandate. But I haven’t heard anything like that lately... and, for me, I feel like it has become more and more meaningful to have this focus. Now I almost feel like...we should have more...that we should only work with the families, at least for the youngest patients here. That...it is amazing to get such a...that you have given us administrative frameworks, and structures and things like that.<br><br>**Facilitates management anchoring**<br>The leadership commitment has definitely been improved. For example, that (NAME of leader) got such an "eye opener" (laughter); That`s what family involvement is about!
**CLINICAL LEVEL**	
**5. Understanding, skills and self-efficacy among mental health professionals**	
Knowledge	**FPE-courses strengthens implementation**<br>One of the most important improvement measures is that we have completed the FPE courses.<br><br>**Access to evidence-based knowledge**<br>In both the groups I have been running, I have enjoyed doing it, and it’s been beneficial to have it there "okay, this is a package, for you to try... This is evidence based, we do this first followed by this"(…). I liked it very much, having this to offer them. So I think it's been great to get trained in that method.

	**Improved handling of ethical dilemmas**<br>I have felt several times that.. Compared to before, I was hiding behind confidentiality, and could not tell ... Well, I felt that I had confidentiality hanging over me, so I did not give information, I wasn't able to n say what I really wanted to say, because ehh .. always having that confidentiality-beast behind me. It's much easier now.
Experience	**Experiencing FPE as a useful method**<br>What I do is very basic things…; I ask them if they (the patients) can tell me about their family. I ask the family members "How has it been for you?" I also provide very basic psychoeducation about stress and vulnerability and the filter, before we talk about it. Well, it's striking me how simple it is and how many people say "I recognise myself in this" and "we should have done this before" or.., especially that recognition part. And that it is something that is possible to get help to work on, right, a more optimistic view. As a therapist, I believe that is an important…feeling, that you have a valuable tool.<br><br>**Experiences family involvement as meaningful work**<br>We are getting feedback from the families that they find it useful. It does something to job satisfaction, quite simply. Satisfaction. When someone ([colleagues]) succeeds… I think: "this has been a good conversation, this is meaningful.<br><br>**Increased self-efficacy**<br>It`s about feeling comfortable and gaining experience in asking questions appropriately. Making the patient feel safe.
**6. Awareness and positive attitudes among mental health professionals towards**	
Relatives	**Sees relatives as a resource**<br>Many of our patients are on medication. Stepping up or reducing medication… many patients want to do that with us, but without relatives knowing anything. But my experience is that it is crucial to bring in the relatives, so that they also know more about it. Based on experiences and family work that I have received feedback on, I assume I will have a different attitude now, if a patient says "No, my relatives cannot know about that".<br><br>**Understands relatives have rights and needs**<br>Now, when the patient does not want us to collaborate with the relatives, I have been even more confident in listening to the relatives about what they have.., about what they already know. And to talk to them. I have been much more confident in that now.<br><br>**Wants to support relatives**<br>There is still so much stigma, there is so much shame and there are so many families isolating themselves, right; "This is kind of my problem, and only my child is experiencing this." So you feel like such a small island in a way…, and "This is the problem we have to deal with on our own", right. Who to talk to? Perhaps they do not have many friends or acquaintances they can talk to about it, because they do not understand it because they do not have similar experiences. Therefore, I think it`s important that we (the professionals) are the ones…. Who know a little more about it… And we can give them hope too, hope for change.

Family involvement	**Increased awareness of the significance of early involvement**<br>It is in a way higher up in the consciousness. I have experienced many times that it is wise, especially to get in touch with relatives in the beginning, because eventually you may very much want to get in contact with them, but then it may be too late in a way, then the patient no longer, or .. There is something about getting in touch (with the relatives) ahead of knowing the person (the patient) so it's not due to us being a little worried about the children, or… Those experiences motivate me, makes me more conscious of it.<br><br>**Increased awareness on long-term benefits**<br>It costs there and then, but hopefully, in the long run, we will get it back in multiples, right. (…) Through many years of experience with patient treatment, we have seen families and relatives in all varieties, right. And it is clear that the more ([patients]) you can help to collaborate with their surroundings (…) the more people you help to live their lives out there .. We should approach the patients holistically, and the relatives and the family are an essential part of their lives.<br><br>**Increased awareness on the national guidelines**<br>P1: It reminds us of the national guidelines for treating psychotic disorders. That it is high up, among the most effective interventions, connecting with the relatives in one way or another. This is useful because that quickly gets forgotten We stick to individual therapy and medication, while forgetting the part of treatment that actually has the best evidence.<br><br>P2: It feels ... sort of professional to say "This is in the guidelines". And it's a good feeling to be able to offer what we consider is the best treatment.
